# Ventricular Tachycardia in Structural Heart Disease

**DOI:** 10.19102/icrm.2019.100801

**Published:** 2019-08-15

**Authors:** Eliany Mejia Lopez, Rohit Malhotra

**Affiliations:** ^1^Cardiac Electrophysiology Department, Cardiovascular Division, Department of Medicine, University of Virginia Health System, Charlottesville, VA, USA

**Keywords:** Ablation, mapping, ventricular tachycardia

## Abstract

Patients with structural heart disease (SHD) are at risk of ventricular tachycardia (VT), which can be difficult to manage clinically. Many treatment options are currently available, but no single approach can be applied with 100% perfect results; often, a combination of therapies is required to achieve good control of ventricular arrhythmias. Coronary artery disease with previous myocardial infarction (MI) is the most common form of SHD presenting with VT, with scar-mediated reentry being the predominant mechanism. Other cardiomyopathies such as arrhythmogenic right ventricular cardiomyopathy, sarcoidosis, Chagas disease, and repaired congenital heart disease can also present in conjunction with ventricular arrhythmias. A thorough analysis of the patient’s history, 12-lead electrocardiogram, and imaging findings are essential for understanding the mechanism and guiding localization of the site of origin of the arrhythmia and the presence of underlying heart disease, which will improve outcomes following catheter ablation if such is indicated. Separately, antiarrhythmic drugs have not been shown to decrease mortality in this patient population but can help to reduce the VT burden and subsequently the need for implantable cardioverter-defibrillator therapy. Unfortunately, most antiarrhythmic agents are negative inotropes, with the possibility of worsening heart failure. This review aims to discuss the current options available for the management of VT in SHD.

## Introduction

Therapeutic options in patients with sustained and recurrent ventricular tachycardia (VT) include antiarrhythmic drugs, transcatheter radiofrequency (RF) ablation, and implantable cardioverter-defibrillator (ICD) therapy. However, many patients with structural heart disease (SHD) have drug-resistant VT or develop an intolerance to the antiarrhythmic drugs used to effectively suppress their arrhythmia, thus requiring the consideration of alternative nonpharmacological methods of therapy. Since the introduction of RF catheter ablation in the 1990s, this technique has been considered effective in both patients with idiopathic VT and those with SHD for the treatment of ventricular arrhythmias.^1–16^

However, it is important to highlight the fact that patients with VT and SHD are a more challenging population given the complexity and heterogeneity of the underlying substrate. The relatively larger size of responsible reentrant circuits, the number of circuits, and variations in depth and scar burden are characteristics that make the performance of this procedure in these patients especially unique and potentially complex.

In the last 20 years, there has been substantial and steady progress made in the treatment of cardiovascular diseases, leading to an overall decrease in patient mortality. However, this has also led to an increased prevalence of heart failure and consequently more individuals living with a risk of sudden cardiac death (SCD). The development of standardized algorithms for the identification and management of patients with a high risk for SCD has increased the use of ICDs for both primary and secondary prevention. As a result, more episodes of VT and ventricular fibrillation (VF) are being diagnosed and treated.

It is well-known that ICD shocks are associated with increased morbidity and mortality^17,18^; therefore, transcatheter ablation for the treatment of recurrent VT is an appealing approach for minimizing ICD shock burden in these individuals. Transcatheter ablation of the site of VT origin has been reported to result in a moderate level of success with suppressing recurrent VT.^14–16,19,20^

In this article, we review the pathophysiology, mechanism, and substrates of ventricular arrhythmias in patients with SHD and discuss the indications for and the role of catheter ablation in this population.

## Pathophysiology of ventricular tachycardia

The sole presence of SHD, either of an ischemic or nonischemic nature, increases the risk of developing arrhythmias and SCD. However, ischemic cardiomyopathy (ICM) and non-ICM (NICM) have different anatomic substrates and electrophysiological characteristics. It is well-described that scar in ICM is often accessible from the endocardium. On the other hand, scar in the nonischemic population is often patchy and located in the midmyocardium or epicardium, making the performance of an invasive procedure more challenging and the scar less accessible. Notably, the mechanisms of scar-mediated reentry form the conceptual basis for the mapping and ablation of VT.

Following a myocardial infarction (MI), infarcts may lead to arrhythmias through different mechanisms. The infarcted myocardium becomes more susceptible to early afterdepolarizations, resulting in triggered activity.^21^ In addition, the different electrophysiologic properties of the healthy myocardium, dense scar, and border zone, respectively, give rise to areas of functional heterogeneity, leading to circus movements and reentry.^22,23^ This heterogeneity can influence the normal behavior of myocyte action potential duration (APD) restitution. Spatial differences in APD and conduction velocity alternans may favor the occurrence of a reentry mechanism.^24^ Furthermore, there are adaptive and maladaptive changes in cardiac autonomic innervation that also predispose to VT. Sympathetic and parasympathetic efferent inputs to local circuit neurons are increased, whereas afferent inputs from infarcted tissue are decreased relative to those from the border zone and normal tissue; this results in heterogeneity of autonomic innervation, contributing to the arrhythmogenic substrate.^25–28^

Multiple pathways can often coexist in areas of dense scar and manifest as multiple VTs of different morphologies. These areas of slow conduction are desirable targets for ablation. Multiple studies performed in canine models,^29–35^ involving mapping in humans,^36–39^ and considering analyses of electrophysiologic study (EPS) results^7,40–44^ provide strong evidence that VT arises from chronic MIs due to reentry and areas of slow conduction. During catheter mapping, areas of slow conduction are identified by long, fractionated electrograms and long S–QRS delays during pacing.

In addition to scar-related reentry, some post-MI patients may present with ventricular arrhythmias related to the Purkinje system. Following MI, triggered activity due to delayed afterdepolarizations from surviving Purkinje fibers situated along the scar border may provoke focal premature ventricular complexes (PVCs) that trigger polymorphic VT/VF.^45–49^ Catheter ablation is possible for the treatment of these various Purkinje-related ventricular arrhythmias.

Scar-related VT is best described in post-MI patients, but the same mechanism is also suggested for any other presentation of cardiomyopathy associated with fibrosis and scar that may cause reentry **(Table 1)**.

## Cardiac imaging in VT

Multimodality imaging techniques play an integral role in determining the underlying substrate for VT. The accurate characterization of the underlying etiology of VT using cardiac imaging has an important impact on therapeutic decisions. According to current guidelines, transthoracic echocardiogram (TTE) and coronary angiogram remain the first-line diagnostic approaches in patients presenting with VT. In recent years, advances in imaging technology have enabled the characterization of the structural arrhythmogenic substrate in patients with VT with increasing precision.^50^

Cardiac magnetic resonance (CMR) imaging is one important tool that has become widely regarded as the gold standard for imaging of the structural VT substrate. CMR imaging provides excellent soft-tissue characterization, detailed assessments of morphology and function, and visualization of myocardial scar with a high degree of precision based on the measurement of the signal intensity of late gadolinium enhancement (LGE).

However, this imaging technique is not without potential limitations in patients with device implants, which is particular in patients with VT.^51–54^ The development of new techniques such as wideband LGE CMR imaging has increased the deployment of CMR imaging in this population because of the removal of image artifacts and more comprehensive evaluation of myocardial scar.^55,56^ All of these new advances increase the utility of CMR imaging, providing valuable information prior to invasive electrophysiologic procedures.

The role of cardiac imaging has expanded from a largely diagnostic tool to an adjunctive tool to guide interventional approaches for the treatment of VT. Mapping systems are in development that allow for the integration of CMR imaging data to aid in catheter ablation. Separately, invasive and noninvasive imaging techniques, often used in combination with one another, have made it possible to integrate both structural and electrophysiological information during VT ablation procedures. An important area of current development is the use of noninvasive imaging techniques based on body surface electrocardiographic mapping to elucidate the mechanisms of VT. In the future, these techniques may provide a priori information on the mechanisms of VT in patients undergoing interventional procedures.

## Catheter ablation for ventricular tachycardia in structural heart disease

In patients with SHD, catheter ablation is used as an adjunctive therapy to ICD implantation when antiarrhythmic drugs are either ineffective or associated with side effects. As such, multiple studies to date have analyzed the role of adjunctive catheter ablation for the secondary prevention of ventricular arrhythmias.

Recently, the Ventricular Tachycardia Ablation Versus Escalation of Antiarrhythmic Drugs (VANISH) trial demonstrated that, in patients with SHD from ICM, an ICD, and recurrent VT despite the use of antiarrhythmic drugs, there was a significantly lower rate of the composite primary outcome. Specifically, the composite primary endpoint of death, VT storm, or appropriate ICD shock was reduced by 28% with ablation [hazard ratio (HR): 0.72; 95% confidence interval (CI): 0.53–0.98; p = 0.04], highlighting the importance of catheter ablation in recurrent VT.^57^

Another important multicenter, randomized trial was the Substrate Mapping and Ablation in Sinus Rhythm to Halt Ventricular Tachycardia (SMASH-VT) study, which evaluated patients with prior MI presenting with spontaneous VT/VF who underwent ICD implantation alone or in combination with catheter ablation. Catheter ablation resulted in a 65% reduction in the rate of appropriate ICD therapy.^19^

Similarly, the Ventricular Tachycardia Ablation in Coronary Heart Disease (VTACH) study randomized patients with a prior MI, a left ventricular ejection fraction of less than 50%, and hemodynamically stable VT to either ICD implantation alone or ICD implantation and ablation. The primary endpoint was time to first recurrence of VT or VF. The time to first recurrence of VT or VF was significantly longer in the ablation group versus in the control group (median: 18.6 months versus 5.9 months). The freedom from VT/VF was 47% in the ablation group and 29% in the control group (HR: 0.61; 95% CI: 0.37–0.99; p = 0.045).^20^

**Table 2** offers a full description of the four randomized clinical trials comparing catheter ablation and conservative therapy. These studies have purported a reduction in the rate of appropriate ICD therapy of between 50% and 65%.

The 2009 European Heart Rhythm Association and Heart Rhythm Society expert consensus statement on the catheter ablation of ventricular arrhythmias recommends catheter ablation in the following scenarios:

1. In patients with sustained monomorphic VT that recurs despite antiarrhythmic drugs or in cases when drugs are not tolerated
2. In bundle-branch reentrant or interfascicular VT
3. For control of incessant sustained monomorphic VT or VT storm not resulting from a reversible cause
4. In patients with frequent PVCs or nonsustained or sustained VT in the setting of ventricular dysfunction
5. In recurrent sustained polymorphic VT and VF that are refractory to antiarrhythmic agents and thought to be amenable for ablation
6. In patients with sustained monomorphic VT despite class I/III antiarrhythmic drug use with prior MI and an LV ejection fraction of more than 30% and as an alternative to antiarrhythmic drugs for hemodynamically tolerated sustained monomorphic VT due to prior MI and LVEF of more than 35%

In addition, the expert consensus statement indicates that catheter ablation of ventricular arrhythmias may be considered prior to ICD implantation in certain patients with frequent PVCs or VT and tachycardia-induced cardiomyopathy, as LV function may improve in these individuals, consequently decreasing the risk of SCD and obviating the need for an ICD.

## Ablation techniques

**Figure 1:**
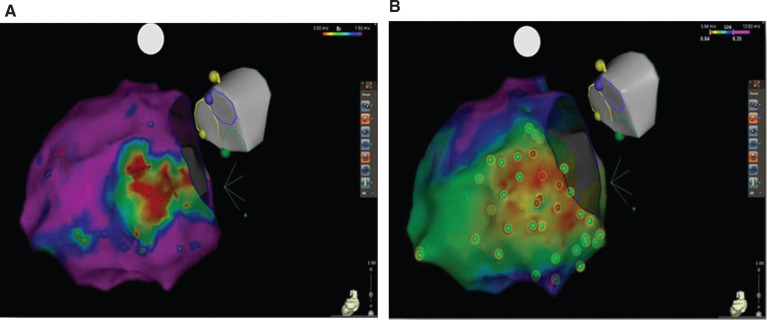
**A:** Electroanatomic bipolar map with evidence of posterior basal endocardial scar, defined by a scar threshold of less than 1.5 mV. **B:** Unipolar endocardial map showing a larger scar area with a low-voltage area (< 8.3 mV). Images courtesy of mages courtesy of Andrew E. Darby, MD, of the University of Virginia.

The catheter ablation of SHD involves the characterization of clinical VTs, the delineation of the arrhythmic substrate, and the radiofrequency ablation of the arrhythmic tissue. Diverse strategies have evolved into being that incorporate imaging technology and electroanatomic mapping to improve the characterization and our understanding of various arrhythmogenic scar substrates **(Figure 1)**. Other approaches include improving the identification of surrogates for critical sites in sinus rhythm, pacemapping, entrainment, involving hemodynamic support devices to increase the duration of mapping in VT, and the performance of more comprehensive and extensive ablation of three-dimensional scar substrates from both the endocardium and epicardium. This review describes the different ablation strategies available and the current supporting evidence.

### Pacemapping and entrainment in stable ventricular tachycardia

**Figure 2:**
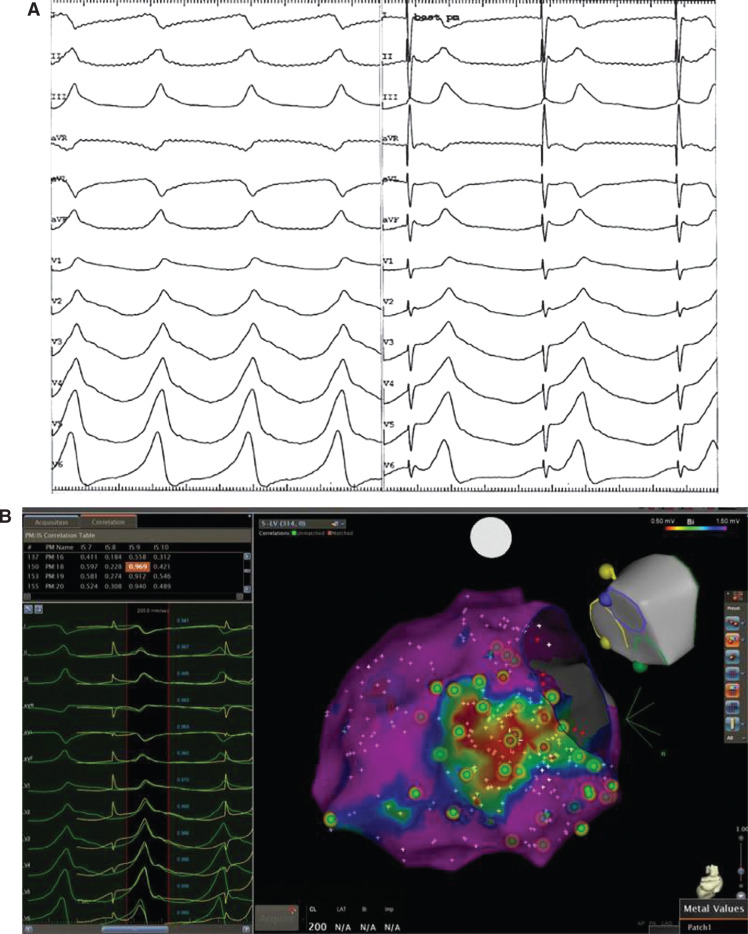
**A:** Example of pacemapping with evidence of a 97% pacemap in all 12 leads. **B:** The best pacemap site is highlighted in the middle of the scar.

Here, the high-density delineation of scar is first performed and regions of slow conduction, as evidenced by fractionated or late potentials, are tagged. Pacemapping is performed to assess the relationship of the pace morphology with the targeted VT. Pacemapping can be an effective method to focus ablation toward specific regions within scar that exhibit a morphologic match to the targeted VT. Pacemaps with stimulus–QRS delays (>40 ms) are more specific, as the conduction slowing out of scar and sites that exhibit multiple QRS morphologies may suggest a common conducting channel.^58,59^ The initiation of VT during pacemapping (ie, pace-mapped induction) can be seen when pacing from an isthmus, and successful termination is seen in more than 90% of cases with ablation at these sites **(Figure 2)**.

### Entrainment

While multiple methods are relied on to accurately localize VT, 12-lead electrocardiogram analysis and imaging are those that are most commonly used. Entrainment maneuvers are also frequently employed. The main principles of entrainment between areas of postinfarct fibrosis were popularized by Stevenson et al.^7^ In this model, the circuit contains an entrance; central isthmus; and exit, which represents the breakout site that results in the formation of the QRS. Regions of the circuit not constrained by scar are termed outer loop sites and regions that are proximal to the entrance, while those constrained by scar are termed inner loop sites. Bystander sites are ineffective sites for ablation and may be remote or attached to the circuit.

**Figure 3:**
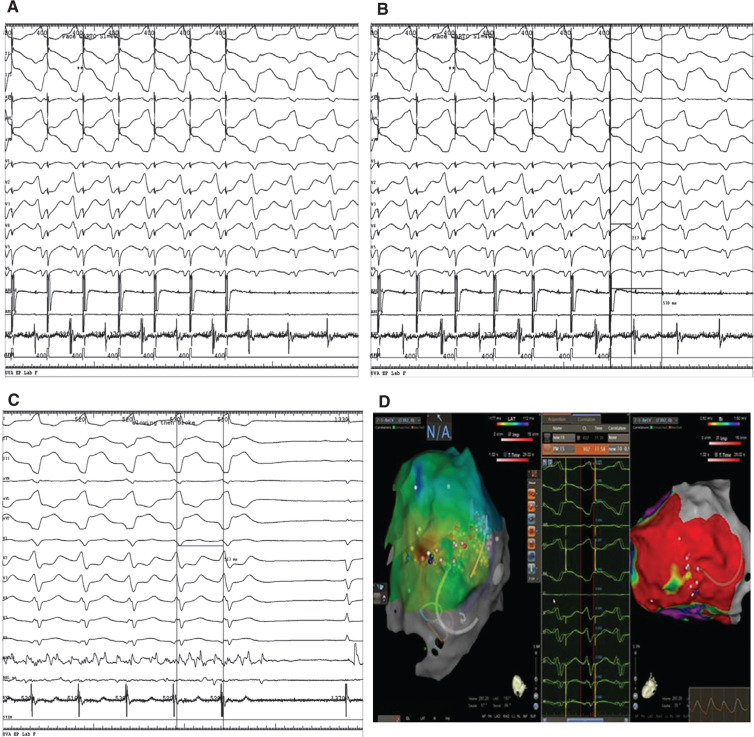
**A:** Entrainment of ventricular tachycardia, showing concealed fusion. **B:** Entrainment with a PPI TCL of ~30 ms to 40 ms with stim-QRS~EGM-QRS. Stim-QRS in 30% to 70% of the TCL. **C:** Slowing and then termination of the tachycardia after RF ablation. **D:** Electroanatomic maps showing early activation areas and areas of scar on bipolar mapping on the right.

The postpacing interval (PPI) is equal to or within 30 ms of the tachycardia cycle length (TCL) at any site that is in the reentrant circuit. Concealed fusion is seen in regions constrained within scar, such that antidromic capture and collision with the orthodromic wavefront occurs entirely within the circuit. The stimulus to QRS exceeds the electrogram-to-QRS interval at bystander sites attached to the circuit. The electrogram-to-QRS interval approximates proximity to the circuit exit, where less than 30% of the TCL is distal, 30% to 70% is central, and more than 70% is proximal within the isthmus **(Figure 3)**. Importantly, the ideal site for ablation within a central isthmus is a mid-diastolic potential during VT that exhibits concealed fusion with a PPI that is equal to the TCL.

Unfortunately, entrainment mapping has multiple limitations. This approach can only be deployed in patients with hemodynamically stable VT; it may overestimate circuit size; and, occasionally, decremental conduction properties of the scar tissue can alter the PPI during the performance of pacing maneuvers. Therefore, other strategies such as voltage-based mapping during sinus rhythm have evolved.

### Sinus rhythm strategy

In patients with unmappable/unstable VT, a sinus rhythm strategy approach is often performed. An accurate and detailed scar characterization is needed for the delineation of border zones and the identification of abnormal electrograms within scar.

This technique uses primarily electroanatomic mapping, which has become an essential tool for optimizing the mapping of VT and guiding ablation lesions. Using a threshold of less than 1.5 mV for low voltage, an arbitrarily defined dense scar threshold of 0.5 mV, and a scar border zone of 0.5 mV to 1.5 mV (when using bipolar electrograms with a 4-mm-tip electrode), the surface voltage map can be displayed as a three-dimensional reconstruction. Endocardial unipolar voltage mapping can also be useful in predicting the epicardial arrhythmia substrate. Unipolar endocardial mapping provides a larger field of view and offers valuable information about the presence or absence of the epicardial substrate as well as the need to pursue epicardial mapping and ablation. Previous studies have employed a unipolar voltage cutoff of 8.27 mV in normal hearts **(Figure 1)**.^60^

The placement of linear lesions guided by electroanatomic mapping was first described in 2000 by Marchlinski et al. in patients with VT previously designated as “unmappable.”^61^ Higher mapping densities can be achieved with multielectrode catheters and have been shown to improve the identification of late potentials in regions of heterogeneity.^62^ Multielectrode mapping can expedite VT ablation using commonly employed techniques including pacemapping, activation, and entrainment mapping and may constitute a more sensitive method to confirm the abolition of late potentials.^63^

**Figure 4:**
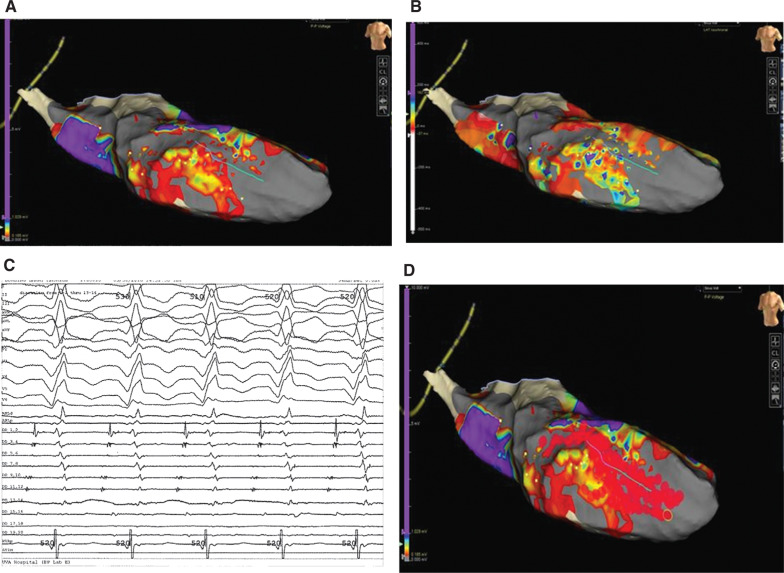
**A:** Sinus rhythm mapping of a patient with ICM showing large and dense anterior scar on bipolar maps (low voltage < 0.5 mV). Note the evidence of a central channel or isthmus after lowering the voltage threshold to 0.185 mV. **B:** Activation map showing areas of isochronal crowding in the isthmus that correspond to low-voltage areas. **C:** Late diastolic potentials when mapping with the Livewire™ duodecapolar catheter 2-2-2- (Abbott Laboratories, Chicago, IL, USA) in sinus rhythm. **D:** Scar dechanneling ablation was performed with noninducibility at the end of the study. Images courtesy of Andrew E. Darby, MD, of the University of Virginia.

Ablation performed using a voltage-based approach requires the precise and accurate definition of the border zones with the identification of channels and isthmuses. This is usually performed by lowering the voltage-dense scar threshold of 0.5 mV, which enables us to identify areas of less healthy tissues but still with some conduction properties functioning as channels or isthmuses that are thus potentially responsive for reentry **(Figure 4)**.

Previous studies have suggested that VT induction and mapping can prolong the procedure, increase radiation exposure, and enhance the need for electrical cardioversion without significantly improving acute results and long-term ablation outcomes in comparison with substrate ablation alone.^64^

Multiple ablation techniques have been used to perform substrate modification, such as targeting core isolation or late potentials (LPs), addressing local abnormal ventricular activities (LAVAs), promoting homogenization of the scar, and performing scar dechanneling. Despite each having a different ablation technique and goal, all have the common end point of inducibility and are performed in sinus rhythm. These approaches are expanded upon henceforth.

***Core isolation.*** Tzou et al. first reported on the technique of core isolation in 2015 when they elegantly described that the core isolation area incorporates critical VT circuit elements.^65^ To achieve core isolation, the first step is to isolate the dense scar (< 0.5 mV) by performing circumferential ablation. The border zone (1.0 mV) is targeted if proven to be part of the reentrant circuit consistent with the isthmus, entrance, or exit sites by way of pacing/entrainment maneuvers. A successful core isolation is defined by noninducibility at a pacing output of 20 mA at a point 2 ms from multiple sites within the core isolation area. The goal of this ablation strategy is to cover the entire scar with ablation lesions targeting abnormal electrograms. This strategy is limited by scar size.

***Late potentials.*** The identification of locally uncoupled and delayed local electrograms or LPs is an important strategy during mapping in sinus rhythm. LPs are defined as any type of electrogram with a duration that extends beyond the end of the surface QRS interval.^66^ These represent areas of slow conduction within scar that serve as the requisite substrate for reentry. Delayed and isolated LPs have been shown to have specificity for induced VTs and ablation aimed at eliminating these abnormal electrograms improves clinical freedom from VT.^67,68^

**Figure 5:**
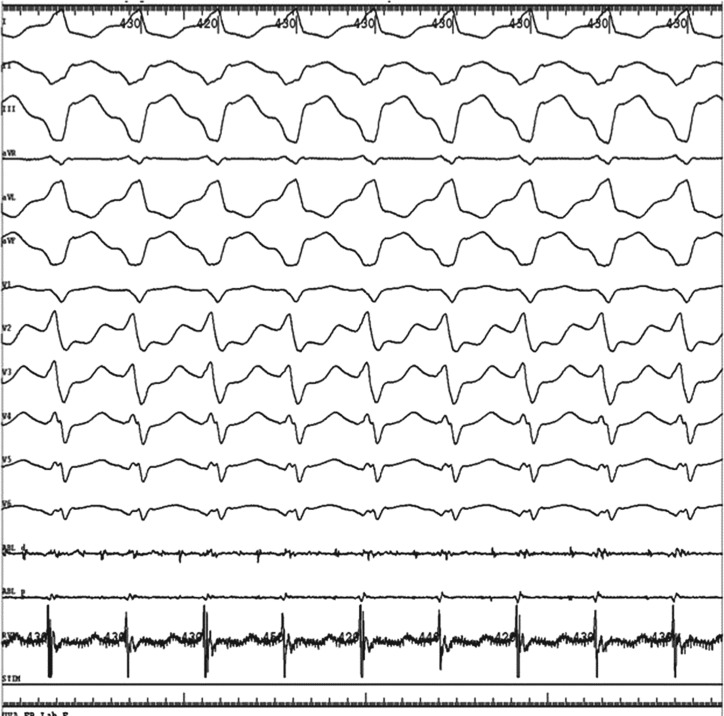
Patient in stable VT. Note the LAVA on the ablation catheter with fractionation and multiple components separated by very-low-amplitude signals. Image courtesy of Andrew E. Darby, MD, of the University of Virginia.

***Local abnormal ventricular activities.*** LAVAs were first described by Jais et al.^69^ and defined as sharp, high-frequency ventricular potentials occurring anytime during or after the far-field ventricular electrogram in sinus rhythm or before the far-field ventricular electrogram during VT that sometimes display fractionation or double or multiple components separated by very-low-amplitude signals or an isoelectric interval and which are not well-coupled to the rest of the myocardium. These signals are considered to be indicative of local activity coming from the pathologic tissue. The use of multipolar catheters has facilitated the identification of these low-amplitude potentials, thus improving outcomes. Evidence suggests that the elimination of LAVAs is safe and associated with improved freedom from recurrent VT as compared with standard ablation **(Figure 5)**.^69^

***Homogenization.*** Extensive and diffuse ablation or “scar homogenization” is aimed at eliminating all abnormal electrograms within the entire scar. The elimination of all electrograms has been shown to be more predictive of clinical success than inducibility. However, it may be difficult to achieve such in instances of large scar substrate.^69^ Following substrate ablation, a high-output pacing at 20 mA from within the scar is performed to confirm that no tissue is captured. Targeting earlier LPs via ablation may eliminate or modify the downstream activation of a channel and expedite homogenization strategies.^70,71^

Of note, the results of the Ablation of Clinical VT Versus Addition of Substrate Ablation on the Long-term Success Rate of VT Ablation (VISTA) randomized trial^72^ support the idea that an extensive substrate-based ablation approach is superior to a clinical targeted ablation protocol in patients with ICM and tolerated VT **(Table 2)**.

***Scar dechanneling.*** Scar dechanneling was first described by Berruezo and colleagues.^70,73,74^ This technique consists of the identification of a corridor of consecutive electrograms with delayed components and subsequent ablation of the entrance regions **(Figure 4)**. The entrances were targeted in the border zone with 0.5 mV to 1.5 mV to avoid ablating healthy tissue beyond the scar. Ablation using scar dechanneling has shown promising outcomes.^70,74^

These substrate-based ablation strategies have been associated with higher arrhythmia-free survival rates and long-term outcomes when compared with the standard ablation of VT in patients with SHD^75^; however, these data mostly include ischemic patients and, thus, the data are limited in terms of their applicability in nonischemic patients.

Even though substrate-based strategies have shown good results, beyond-voltage or functional substrate mapping is also gaining popularity. This approach looks for areas of late and slow conduction, identified by isochronal late activation maps (ILAMs). Slow-conduction regions with isochronal crowding propagating into the latest zone of activation may be a promising ablation target for substrate modification. The creation of ILAMs during sinus rhythm may provide complementary and targeted functional information beyond structural voltage displays for scar-related VT and represent a novel strategy for substrate-based VT ablation **(Figure 4)**.^76^

## Epicardial ventricular tachycardia ablation

The indications for epicardial mapping are variable but typically performed after a failed endocardial approach, or in cases where the etiology of cardiomyopathy suggests a high likelihood of epicardial scar, such as in NICM patients.

Different parameters are used during epicardial mapping, with the low-voltage area characterized by a bipolar signal amplitude of less than 1.0 mV.^75^ Endocardial unipolar voltage mapping can also be useful in certain scenarios, including specifically to predict the epicardial arrhythmia substrate, such as in patients with dilated cardiomyopathy where normal left ventricular tissue has been reported to be greater than or equal to 8.27 mV or in those with arrhythmogenic right ventricular dysplasia (ARVC) where normal right ventricular tissue is greater than or equal to 5.5 mV.

Studies of patients with NICM who underwent a combined epicardial–endocardial approach to VT ablation have consistently demonstrated more extensive epicardial voltage abnormalities in comparison with the endocardial surface.^77–79^ Similar observations have been made in patients with hypertrophic cardiomyopathy and ARVC and a combined epicardial–endocardial approach has been indicated to be more effective than an endocardial approach alone in several observational reports.^80,81^

## Endpoints after ventricular tachycardia ablation

The most important endpoint following VT ablation is the achievement of noninducibility. However, while noninducibility has been shown to be predictive for clinical freedom from recurrent VT, this has not uniformly been the case.^82,83^ Inducibility of VT is probabilistic and rarely is reproducibly triggered on command. Furthermore, inducibility can be affected by anesthesia and antiarrhythmic drugs.

Although homogenization is increasingly being performed, an objective measure to demonstrate the complete elimination of abnormal electrograms has not yet been established at this time. The use of multipolar mapping catheters may be beneficial to rapidly remap ablated regions to ensure the abolition of LPs.^84^ In other arrhythmias treated with linear lesions, bidirectional block has been shown to be the most robust electrophysiological endpoint. Potential objective endpoints would require remapping to demonstrate a change in scar propagation that would reflect a line of block or the elimination of the latest regions of activation.

## Conclusions

In the last two decades, there have been significant advancements made in the field of electrophysiology, especially for VT ablation. Our understanding of various scar substrates has improved significantly due to the development of better electroanatomic mapping systems, the use of CMR imaging, the application of multielectrode catheter mapping, and the employment of percutaneous epicardial access. These novel technologies facilitate the accurate delineation of areas of scar using higher-resolution mapping and subsequently improve the outcomes of the procedure in patients with structural heart disease while also significantly reducing the incidence of recurrent ventricular arrhythmias.

More extensive ablation strategies or “homogenization” and ablation in sinus rhythm using novel approaches will continue to evolve and help to improve clinical outcomes. Technological advancements in substrate imaging, mapping, bipolar radiofrequency ablation, intramural needle ablation catheters, and the use of stereotactic radiotherapy for ablation of drug-resistant and recurrent VT will further improve outcomes after ablation.

## Figures and Tables

**Table 1:** Substrate Localization and Distribution in Structural Cardiomyopathy

	Ischemic Cardiomyopathy	Nonischemic Dilated Cardiomyopathy	ARVC/D	Cardiac Sarcoidosis	HOCM	Chagas Disease
Substrate	Subendocardial	Midmyocardial/epicardial	Subepicardial	Nodular, circumferential, subepicardial, and subendocardial	Subendocardial	Epicardial > endocardial
Scar distribution	Focal	Patchy	Patchy		Focal anterior and posterior RV insertion points	Patchy
Scar location	Coronary distribution	Basal anteroseptal and inferolateral LV regions	RV > LV; triangle of dysplasia*	Basal and mid-interventricular septum	LV apex and aneurysmal areas	Inferolateral and apical LV
Ablation approach	Endocardial	Endocardial/epicardial	Endocardial/epicardial	Endocardial > epicardial	Endocardial	Epicardial and endocardial

ARVC/D: Arrhythmogenic right ventricular cardiomyopathy/dysplasia; HOCM: hypertrophic obstructive cardiomyopathy; LV: left ventricle/ventricular; RV: right ventricle/ventricular.

*Triangle of dysplasia: RV subtricuspid areas, apex, and infundibulum.

**Table 2:** Randomized Controlled Trials of Prophylactic Catheter Ablation in SHD Patients

Trial	Study Group		Number of Patients Randomized	Ablation Strategy	Follow-up Time	Primary Endpoint	Outcomes	
Reddy et al. 2007^19^	•	ICM	128	Substrate-based with mapping in sinus rhythm	22.5 ± 5.5 months	Survival free from ICD shocks	•	65% reduction in the rate of appropriate ICD therapy
	•	Arms: ablation vs. ICD alone						
Sapp et al. 2016^57^	•	ICM	259	Activation mapping targeting clinical VT vs. substrate-based approach	27.9 ± 17.1 months	Composite of death, VT storm, or appropriate ICD shock	•	Appropriate ICD shock was reduced by 28% with ablation (HR: 0.72, 95% CI: 0.53–0.98; p = 0.04)
	•	Arms: ablation vs. escalated antiarrhythmic drugs						
Di Biase et al. 2015^72^	•	ICM	118	Clinical ablation with activation and entrainment mapping vs. substrate modification	12 months	Recurrence of VT	•	Extensive substrate-based ablation was superior to clinical target ablation
	•	Arms: clinical target ablation vs. substrate-based ablation						
Tanner et al. 2010^83^	•	ICM	110	Entrainment mapping ± pacemapping ± substrate modification	22.5 months	Time to first recurrence	•	18.6 months in the CA group vs. 5.9 months in the control group
	•	Arms: ablation vs. ICD alone					•	Freedom from VT/VF was 47% in the CA group and 29% in the control group (HR: 0.61, 95% CI: 0.37–0.99)
Dinov et al. 2014^85^	•	ICM and NICM patients undergoing ablation	227	Activation and entrainment mapping with elimination of all clinically and nonclinically stable MMVT	12 months	Survival free of VT	•	No difference in short-term outcomes
							•	VT-free survival in NIDCM was 40.5% vs. 57% in ICM
Kuck et al. 2017^86^	•	ICM	111	Entrainment mapping ± pacemapping ± substrate modification	2.3 ± 1.1 years	Time to first recurrence of VT/VF	•	No difference in the time to first recurrence
	•	Arms: ablation vs. ICD alone					•	CA resulted in a ; 50% reduction in total ICD interventions in the ablation group

CA: catheter ablation; CI: confidence interval; HR: hazard ratio; ICD: implantable cardioverter-defibrillator; ICM: ischemic cardiomyopathy; MMVT: monomorphic ventricular tachycardia; NICM: nonischemic cardiomyopathy; VT: ventricular tachycardia; VF: ventricular fibrillation.
